# Association of hearing impairment with chronic kidney disease: a cross-sectional study of the Korean general population

**DOI:** 10.1186/s12882-015-0151-0

**Published:** 2015-09-16

**Authors:** Young Joon Seo, Sang Baek Ko, Tae Hyung Ha, Tae Hoon Gong, Jeong Pyo Bong, Dong-Joon Park, Sang Yoo Park

**Affiliations:** Department of Otorhinolaryngology, Yonsei University Wonju College of Medicine, Wonju, Korea; Department of Occupational and Environmental Medicine, Yonsei University Wonju College of Medicine, Wonju, Korea

## Abstract

**Background:**

We aimed to evaluate the association between hearing impairment and the prevalence of chronic kidney disease (CKD) in the largest population-based cross-sectional study to date.

**Methods:**

This cross-sectional study was based on the Korean National Health and Nutritional Examination Survey (KNHANES). It included 5226 participants ≥19 years of age whose estimated glomerular filtration rate (eGFR) and hearing threshold had been measured. We diagnosed CKD as an eGFR <60 mL/min/1.73 m^2^. The participants were also evaluated for the presence of other contributing factors related to kidney dysfunction. We divided the participants at the 40-dB threshold into hearing-impairment and no-hearing-impairment groups, using the average threshold of all six frequencies (500, 1000, 2000, 3000, 4000, and 6000 Hz) for both ears.

**Results:**

The odds of hearing impairment was 1.25 times higher (95 % confidence interval: 1.12–1.64, *p*-value < 0.001) in participants with an eGFR <60 mL/min/1.73 m^2^ than in those with an eGFR ≥60 mL/min/1.73 m^2^ after adjustments for age, sex, smoking, alcohol, body mass index, diabetes mellitus, hypertension, dyslipidemia, and microalbuminuria. Among the risk parameters of CKD associated with hearing impairment, linear regression analysis adjusted for age and sex determined that each increase of serum creatinine or blood pressure was positively associated with an increase in hearing threshold (*p*-value < 0.01).

**Conclusion:**

The odds of hearing impairment were greater with lower eGFR than with normal eGFR. Individuals with CKD were more likely to also have hearing impairment. We recommend screening the hearing of patients with CKD to provide earlier identification of hearing impairment and earlier intervention, thereby preventing progression of hearing impairment and providing appropriate treatment strategies.

## Background

The association between chronic kidney disease (CKD) and hearing impairment was first reported more than 80 years ago by Alport [1], who described a case of familial kidney disease related to hearing impairment. With CKD considered to be a major public health concern due to the increase in prevalence in adults, some studies have subsequently suggested the possibility of a link between the ear and the kidney [2–4]. However, all of the current evidence is derived from small observational studies of patients with CKD or samples of patients with renal failure. To date, only a small number of large population-based studies have assessed the association between nonsyndromal CKD and hearing impairment. Vilayur et al. [5] reported that moderate CKD was associated independently with hearing impairment in a large community-based study after adjusting for age, sex, and other contributing factors.

The kidney and stria vascularis of the cochlea share physiologic, ultrastructural, and antigenic similarities that could explain the link between CKD and hearing impairment [6]. The similarity between the stria vascularis and the renal tubules could account for why most nephrotoxic medication is ototoxic in nature [2]. Nevertheless, these reported connections and analogies between the ear and the kidney did not deserve to be formed into an established hypothesis due to insufficient evidence. Given that the complication of hearing impairment in patients with CKD is multifactorial, it should be considered in hearing impairment studies of patients with CKD that many multiple contributing factors have been hypothesized to cause hearing impairment, including use of ototoxic medications, hypertension, and diabetes, as well as electrolyte disturbances and hemodialysis itself [7–9].

We conducted a large cross-sectional analysis on the associations between hearing impairment with CKD in a representative sample of the adult Korean population from the Korean National Health and Nutritional Examination Survey (KNHANES). The aim of this study was to confirm whether CKD could be an independent contributing factor for hearing impairment and then to identify which CKD-related risk parameters were important contributing factors for hearing impairment among people with nonsyndromal CKD.

## Methods

### Study population

The KNHANES is a cross-sectional and nationally representative survey composed of a health questionnaire survey, health examination, and nutrition survey. The KNHANES V was conducted from 2010 to 2012 using a stratified, multistage probability sampling design, and we used recent data from the third year (2012) of the KNHANES V in this study. In the 2012 survey, 10,069 individuals participated in health interviews, health examination surveys, and nutritional surveys. A trained otologist performed face-to-face interviews with each patient to collect a comprehensive medical history that included information about the presence of syndromes related to kidney disease and hearing. We analyzed the data of participants aged ≥19 years. Participants with any of the following conditions were excluded: (1) history of treatments for renal failure or syndromes associated with kidney dysfunction (Alport syndrome, nephrotic syndrome, etc.); (2) history of chronic otitis media; (3) external- or middle-ear problems on physical examination; (4) history of noise-induced hearing impairment; or (5) incomplete hearing results. Using the questionnaire for quantitative evaluation (history of occupational noise exposure, duration of noise exposure, use of hearing protective devices, and exposure to instantaneous environmental noise), 101 patients with noise-induced hearing impairment were excluded, as noise-induced damage of the cochlea could create bias when evaluating the independent role of CKD for hearing. Exposure to occupational noise was determined according to whether the participant had worked in a location with loud machines for ≥3 months. A loud noise was defined as whether the participant had to raise his or her voice to hold a conversation.

A total of 5226 participants who underwent audiometric testing were included in this cross-sectional analysis. The Korea Centers for Disease Control and Prevention approved the study protocol, and written informed consent was obtained from all participants before the study began.

### Physical examination and pure-tone audiometry

Ear examinations were performed by ear, nose, and throat residents using a 4-mm 08 endoscope (Xion GmbH) and the ML 150 vision system (JRMed Trade). The air-conduction hearing threshold was measured by well-trained examiners in a double-walled soundproof booth using an automatic audiometer (SA-203; Entomed) at 500, 1000, 2000, 3000, 4000, and 6000 Hz. In this study, the pure-tone audiogram average was calculated as the average threshold of all six frequencies. We included the 6000-Hz threshold to detect early presbycusis [10]. We also calculated average thresholds at low-frequency (500 and 1000 Hz), mid-frequency (2000 and 3000 Hz), and high-frequency (4000 and 6000 Hz) ranges. After finding that the average hearing threshold of the left ear (18.0 ± 17.1 dB) appeared to be similar to that of the right ear (17.6 ± 16.9 dB), we used the average result of both ear data with CKD for a stricter analysis. Participants were classified as having a hearing impairment if the average hearing threshold exceeded 40 dB in each ear [11].

### Data collection of possible contributing factors for hearing impairment

Information on age, sex, residence area (urban or rural), educational status, smoking exposure, alcohol drinking, occupation, and other variables in the KNHANES were obtained through a self-reported survey. Body mass index (BMI; kg/m^2^) was calculated by dividing weight by the height squared. Smoking history was positive if subjects had smoked >5 pack-years during their entire lives. Alcohol drinking was indicated by the consumption of at least 30 g/day over the last year. Seoul (the capital city of South Korea) and six other metropolitan cities were grouped as urban areas, while the remaining regions were grouped as rural areas. Educational status was divided into having or not having attended college. Histories of explosive or occupational noise exposure were considered positive or negative according to the subjects’ recall. An explosive noise was defined as a sudden loud noise such as an explosion or gunshot. Exposure to occupational noise was determined according to whether the participant had worked in a location with loud machines for ≥3 months. A loud noise was defined as the participant having to raise his or her voice to hold a conversation. Subjects were asked if they had ever been diagnosed with diabetes, hypertension, dyslipidemia, thyroid dysfunction, or chronic renal failure by a doctor.

Blood samples were taken after an 8-hour fast during the period in which this survey was performed. The glycosylated hemoglobin (HbA1_C_), total cholesterol, triglyceride, high-density lipoprotein cholesterol, low-density lipoprotein cholesterol, blood urea nitrogen (BUN), and creatinine concentrations were measured using a Hitachi 7600–110 chemistry analyzer (Hitachi, Tokyo, Japan). Fasting blood sugar was measured using an automated analyzer with an enzymatic assay (Pureauto S GLU; Daiichi, Tokyo, Japan). Urinary albumin was measured using a turbidimetric immunoassay (Automatic Analyser 7600; Hitachi). To compare the amount of albumin in each sample against the concentration of creatinine in the spot urine sample, the albumin/creatinine ratio (ACR) was used; microalbuminuria was defined as ACR ≥3.5 mg/mmol (female) or ACR ≥2.5 mg/mmol (male). The estimated glomerular filtration rate (eGFR) was determined using the CKD-EPI formula: eGFR (mL/min/1.73 m^2^) = 141 × min(Scr/κ,1)^α^ × max(Scr/κ,1) ^−1.209^ × 0.993^Age^ × 1.018, if female (Scr = serum creatinine, max = the maximum of Scr/κ or 1, κ = 0.7,α = −0.329 if female) [12]. We focused on stage III CKD, which was defined for the purposes of this study as eGFR <60 mL/min/1.73 m^2^.

### Statistical analysis

We used the stratification variables and sampling weights designated by the Korean Centers for Disease Control and Prevention. Continuous data are expressed as means and standard deviation, while categorical data are expressed as percentages. An independent *t*-test was used to compare continuous variables and the chi-square test was used to compare categorical variables.

Demographic characteristics were analyzed according to the hearing threshold (<40 or ≥40). Logistic regression analysis was applied to examine whether hearing impairment was associated with CKD and was fitted with increasing degrees of adjustment: a model adjusted for age (Model 1) and a model adjusted for age, sex, smoking, alcohol, BMI, DM, HTN, dyslipidemia, and microalbuminuria (Model 2). Linear regression was used to assess the associations between risk parameters related with kidney dysfunction and hearing impairment with adjustment for age and sex. For the multicollinearity diagnosis among variables, variance inflation factors (VIF) were calculated for each variable. The VIF provided an index that could measure how much the variance of an estimated regression coefficient increased due to collinearity. The odds ratios (OR) and 95 % confidence intervals (95 % CI) of the associated factors for reduced eGFR were determined. Values of *P* < 0.05 were considered statistically significant. Statistical analyses were performed using SPSS version 18.0 (SPSS, Inc.).

## Results

**Fig. 1:**
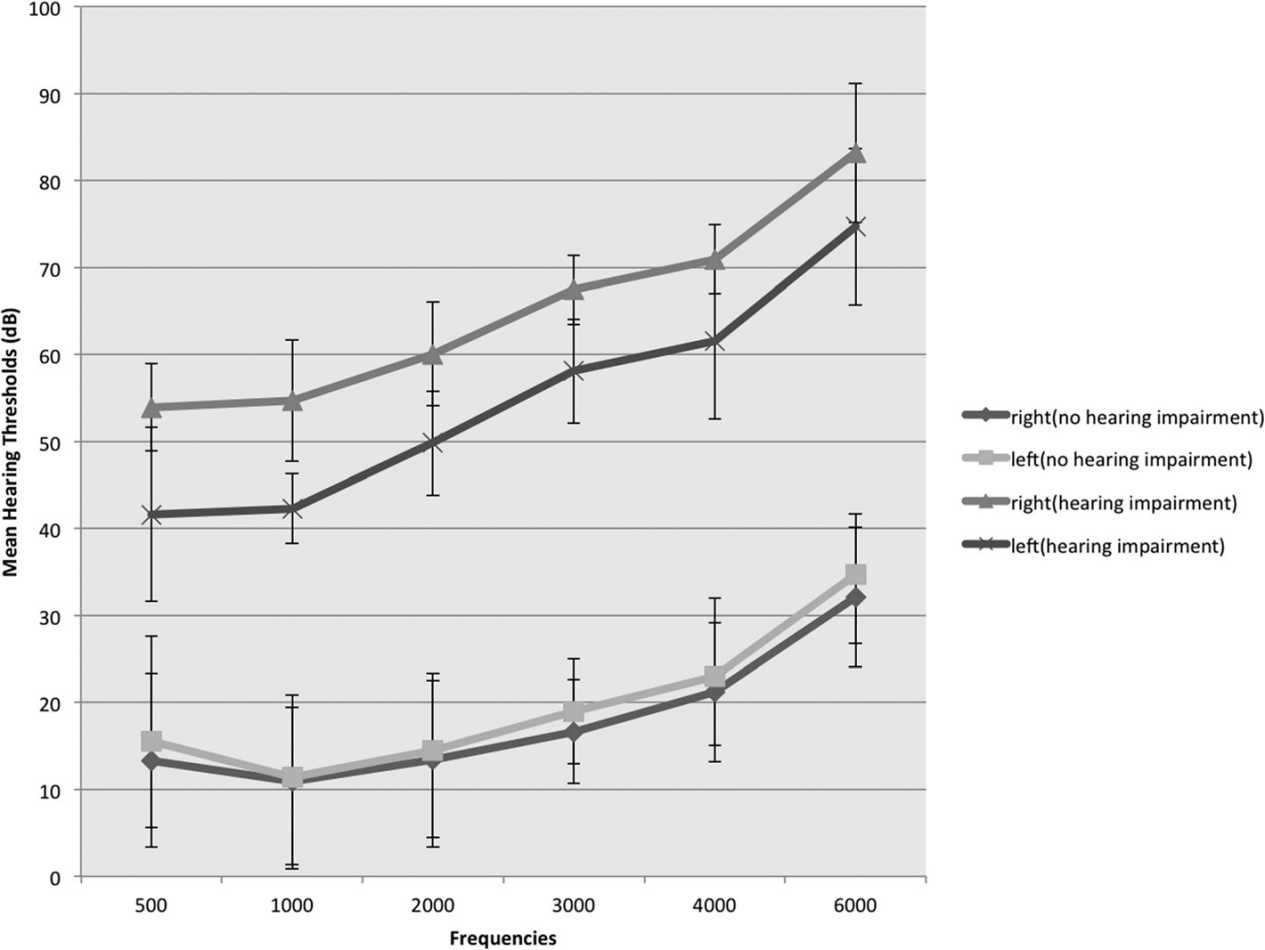
Mean hearing thresholds on both ears in both groups. The hearing thresholds were worse significantly at every frequency in the hearing-impairment group than in the no-hearing-impairment group

The participants’ general and anthropometric characteristics and clinical and laboratory parameters stratified by hearing impairment status are shown in Table 1. The mean ages of the study participants were 49.6 ± 15.7 years (no-hearing-impairment group) versus 70.2 ± 9.9 years (hearing-impairment group). The hearing-impairment group had a worse mean hearing thresholds (i.e. the mean threshold of both ears) than the no-hearing-impairment group (54.6 ± 14.3 vs. 13.8 ± 9.7 dB, respectively), and there were significant differences in hearing thresholds at every frequency (Fig. 1). Without adjusting for other factors, participants with a hearing impairment seemed to be more frequently influenced by hypertension, diabetes, or dyslipidemia (*p*-value < 0.05 for all) than those without. The risk parameters (urine albumin, urine creatinine, serum creatinine, BUN, SBP, and DBP) for kidney dysfunction were worse in the hearing-impairment group than in the no-hearing-impairment group. Regarding eGFR, the key parameter for CKD, 10.1 % of patients with no hearing impairment and 47.1 % of patients with hearing impairments had reduced eGFR (<60 mL/min/1.73 m^2^). The ratios of microalbuminuria between the groups were significantly different; however, the occurrences in both groups were not high (1.3 % vs. 2.9 %).

**Table 1:** Demographic and clinical characteristics of participants stratified by hearing impairment

Characteristics	No hearing impairment (*n* = 4673)	Hearing impairment (*n* = 515)	*p*-value
Age (years), ≥65	49.6 ± 15.7	70.2 ± 9.9	**<0.001**
Sex (male)^a^	1914 (41.0)	253 (49.1)	**<0.001**
BMI (kg/m^2^)	23.8 ± 3.4	23.6 ± 3.2	0.239
Smoking (current)^a^	1845 (38.5)	253 (47.5)	**<0.001**
Alcohol (≥30 g/day)^a^	481 (10.3)	52 (10.1)	0.491
Residence (rural)^a^	2397 (51.3)	241 (46.7)	0.442
Education (>college)^a^	1505 (32.2)	52 (10.1)	**<0.001**
Occupation^a^			0.411
Services and others	518 (11.1)	31 (6.0)	
Industry	411 (8.8)	28 (5.5)	
Agriculture and fishery	313 (6.7)	63 (12.4)	
Hypertension (yes)^a^	682 (14.6)	156 (30.2)	**<0.001**
Diabetes (yes)^a^	439 (9.4)	108 (21.0)	**<0.001**
Dyslipidemia (yes)^a^	715 (15.3)	190 (19.2)	**0.046**
Thyroid dysfunction (yes)^a^	78 (1.6)	17 (3.4)	0.081
HbA1_C_	5.8 ± 0.8	6.01 ± 0.9	**<0.001**
Blood lipid profile			
Total cholesterol	190.3 ± 36.0	189.0 ± 37.5	0.465
HDL cholesterol	52.0 ± 12.6	48.6 ± 11.9	**<0.001**
LDL cholesterol	121.1 ± 34.9	112.8 ± 38.0	0.076
Triglyceride	130.2 ± 98.4	141.3 ± 123.6	0.062
Urine albumin (ug/ml)	230.2 ± 116.1	392.1 ± 119.8	**<0.001**
Urine creatinine (mg/dL)	153.5 ± 86.8	122.1 ± 66.3	**<0.001**
ACR	2.0 ± 1.4	4.1 ± 1.5	**<0.001**
Microalbuminuria^a^	61 (1.3)	15 (2.9)	**0.008**
Serum creatinine	0.83 ± 0.23	0.89 ± 0.27	**<0.001**
BUN	14.3 ± 4.3	16.7 ± 5.2	**<0.001**
eGFR (mL/min/1.73 m^2^)	91.9 ± 26.4	63.6 ± 19.7	**<0.001**
eGFR < 60^a^	472 (10.1)	243 (47.1)	**<0.001**
SBP (mmHg)	118.8 ± 16.6	128.6 ± 17.4	**<0.001**
DBP (mmHg)	75.9 ± 10.4	74.2 ± 10.2	**<0.001**
Mean hearing level (dB)	13.8 ± 9.7	54.6 ± 14.3	**<0.001**

Data expressed as mean ± SD, *p* < 0.05 was set as the significance level, and significant differences between groups are shown in bold. We divided the participants at the 40-dB threshold into the hearing-impairment and no-hearing-impairment groups, using the average threshold of all six frequencies (500, 1000, 2000, 3000, 4000, and 6000 Hz) for both ears

*BMI* body mass index, *HbA1*_*C*_ glycosylated hemoglobin, *HDL* high-density lipoprotein, *LDL* low-density lipoprotein, *UACR* urinary albumin creatinine ratio, *BUN* blood urea nitrogen, *eGFR* estimated glomerular filtration rate, *BUN* blood urea nitrogen, *SBP* systolic blood pressur, *DBP* diastolic blood pressure, *dB* decibel, microalbuminuria defined as ACR ≥3.5 mg/mmol (female) or ACR ≥2.5 mg/mmol (male)

^a^Data are expressed as number (%) unless otherwise indicated

**Fig. 2:**
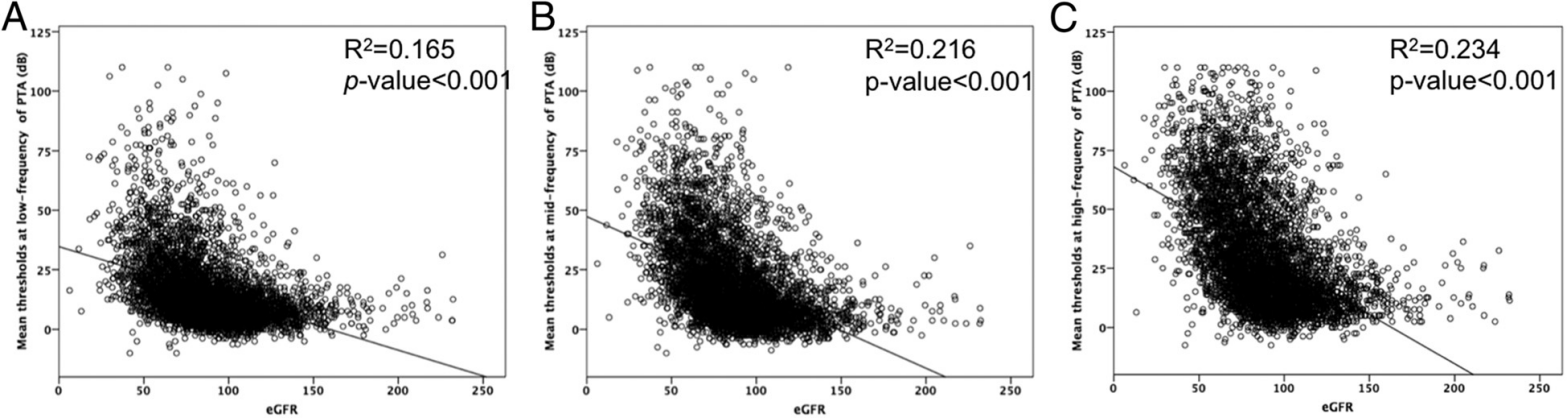
The correlation between eGFR and mean thresholds at low- (**a**), mid-(**b**), and high-(**c**) frequency ranges. All of the three groups had meaningful negative correlations with eGFR values (all *p* < 0.001)

To evaluate which frequencies would be influenced by low eGFR, we divided the frequencies into three groups (low-, mid-, and high-frequency ranges). All of the three groups had meaningful negative correlations with eGFR values (Fig. 2). Furthermore, through each of the low-, mid-, and high-frequency ranges, the participants with low eGFR (<60) had worse hearing thresholds than those with relatively higher eGFR (≥60; Table 2). The difference in mean thresholds for the total frequency range between the groups was significant, similar to those for the three frequency ranges.

**Table 2:** Mean hearing thresholds at each low-, mid-, and high- frequency between the groups according to eGFR <60, adjusted for age

Mean thresholds of PTA			Unadjusted		Adjusted with Age	
	eGFR ≥60	eGFR <60	β (SE)	*p*-value	β (SE)	*p*-value
	(*n* = 4245)	(*n* = 716)				
Low-frequency	13.5 ± 12.3	29.2 ± 18.7	−0.101(0.006)	**<0.001**	−0.032(0.008)	**0.047**
Mid-frequency	16.4 ± 16.1	38.1 ± 20.8	−0.103(0.007)	**<0.001**	−0.078(0.009)	**0.033**
High frequency	27.8 ± 21.3	53.9 ± 22.6	−0.107(0.009)	**<0.001**	−0.047(0.011)	**0.042**
Total frequency	14.9 ± 13.4	33.6 ± 18.9	−0.152(0.006)	**<0.001**	−0.050(0.008)	**0.021**

Data expressed as mean ± SD, *p* < 0.05 was set as the significance level, and significant differences between groups are shown in bold

*eGFR* estimated glomerular filtration rate, low-frequency (500 and 1000 Hz), mid-frequency (2000 and 3000 Hz), and high-frequency (4000 and 6000 Hz) ranges. *β* unstandardized coefficient, *SE* standard error

The prevalence of CKD (eGFR <60 mL/min/1.73 m^2^) in each group also was analyzed using logistic regression analysis with adjustment models (Table 3). For hearing impairment, the OR comparing an eGFR <60 versus ≥60 mL/min/1.73 m^2^ in Model 1 adjusted for age was 1.41 (95 % CI, 1.08–1.83, *p*-value- < 0.001). In Model 2, after adjustment for age, sex, smoking, alcohol, BMI, and parameters of chronic disease, the OR was also significant only for the parameter of eGFR (OR, 1.25; 95 % CI, 1.12–1.64; *p-*value < 0.001).

**Table 3:** Multiple logistic regression analysis between parameter of chronic disease and hearing impairment

	Odds ratio (95 % confidence interval)			
Parameter	Model 1	*p*-value	Model 2	*p*-value
eGFR <60	1.41 (1.08, 1.83)	<0.001	1.25 (1.12, 1.64)^a^	<0.001
HTN	0.87 (0.44, 1.72)	0.097	0.97 (0.46, 2.04)^b^	0.967
DM	1.07 (0.49, 2.33)	0.089	0.77 (0.99, 5.88)^c^	0.161
Dyslipidemia	1.35 (0.14, 12.92)	0.794	0.92 (0.71, 1.19)^d^	0.505
Microalbuminuria	1.00 (0.99, 1.01)	0.187	1.15 (0.57, 2.33)^e^	0.699

Model 1 adjusted for age

Model 2 ^a^adjusted for age, sex, smoking, alcohol, BMI, DM, HTN, dyslipidemia, and microalbuminuria; ^b^adjusted for age, sex, BMI, DM, eGFR <60, dyslipidemia, and microalbuminuria; ^c^adjusted for age, sex, BMI, HTN, eGFR <60, dyslipidemia, and microalbuminuria; ^d^adjusted for age, sex, BMI, DM, HTN, eGFR <60, and microalbuminuria; ^e^adjusted for age, sex, BMI, DM, HTN, eGFR <60, and dyslipidemia

*BMI* body mass index, *eGFR* estimated glomerular filtration rate, *DM* diabetes mellitus, *HTN* hypertension; microalbuminuria, albumin/creatinine ratio (ACR) ≥3.5 mg/mmol (female) or ACR ≥2.5 mg/mmol (male), *CI* confidence interval, *OR* odds ratio

Among the risk parameters of CKD associated with hearing impairment, linear regression analysis adjusted for age and sex determined that each increase of serum creatinine or blood pressure was positively associated with an increasing hearing threshold (*p*-value < 0.01; Table 4). The VIFs of all variables were <2.5, which means that those risk parameters had no significant inter-correlations in this model.

**Table 4:** Multiple linear regression analysis for the mean hearing thresholds (dB) by risk parameters related to kidney dysfunction

	β (SE)	*P* value
Serum creatinine	2.29 (1.01)	<0.01
BUN	0.56 (0.05)	0.08
Microalbuminuria	−0.02 (0.01)	0.76
Urinary creatinine	−0.23 (0.01)	0.27
HbA1_C_	2.08 (0.26)	0.49
SBP	0.31 (0.02)	<0.01
DBP	0.33 (0.03)	<0.01

Values were adjusted for age and sex

*BUN* blood urea nitrogen, *HbA*_*1C*_ glycosylated hemoglobin, *SBP* systolic blood pressure, *DBP* diastolic blood pressure, *β* unstandardized coefficient, *SE* standard error

## Discussion

In this study, we used KNHANES data to investigate the potential association between CKD and hearing impairment in Korean adults. An eGFR <60 mL/min/1.73 m^2^ had a significant independent influence on the hearing thresholds of adults with nonsyndromal CKD. The OR of the hearing impairment (as defined by an average threshold ≥40 dB) was 1.25 times higher in participants with an eGFR <60 mL/min/1.73 m^2^ than in those with an eGFR ≥60 mL/min/1.73 m^2^ after adjustment for age, sex, smoking, alcohol, BMI, DM, HTN, Dyslipidemia, and microalbuminuria. We observed in this study that CKD (defined as an eGFR <60 mL/min/1.73 m^2^) could affect hearing impairment independently. Blood pressure and serum creatinine were found to be positively correlated with hearing impairment among kidney dysfunction–related risk parameters.

Recent research in a large Australian population reported a relatively high prevalence of hearing impairment associated with moderate CKD, and more than half of the participants with CKD had any level of hearing impairment [5]. Others demonstrated that the high frequency thresholds were significantly higher for patients with CKD [2–4]. Morton et al. [3] showed an increasing incidence of hearing impairment in hemodialysis patients that was unrelated to ototoxic drugs. Antonelli et al. [13] reported that 39.1 % of patients with CKD had an abnormal auditory brainstem response compared with 23.9 % of controls. Furthermore, in another prospective study evaluating cochlear functioning in patients (18–45 years old) with varying stages of CKD, 46 % of the participants with later-stage CKD presented with early cochlear dysfunction and subclinical hearing impairment [14]. Our findings are in line with these studies. However, there were significant differences through all of the frequencies on both ears in our study, not only for the high frequency range. This is why we compared the mean thresholds for the total frequency range with eGFR. According to Gelfand’s hearing impairment criteria [11], people who presented with hearing thresholds of ≤25 dB were considered to have normal hearing as indicated by normative data. However, as socially serviceable hearing is within 40 dB and disabling hearing impairment refers to hearing impairment >40 dB [15], we divided the participants at the 40-dB threshold into the hearing-loss and no-hearing-loss groups. This eliminated participants with a hearing threshold of 25–40 dB, making the difference in eGFR clearer at every frequency discriminated.

The pathophysiology between hearing impairment and CKD remains unclear. Various factors can contribute to cochlear dysfunction in patients with CKD, and a combination and interaction of several factors could result in reduced cochlear functioning in patients with CKD. The cochlea and the kidney show noteworthy similarities such as membranous structures, the central role of ciliated epithelial cells, and tubular organization [6]. One research group hypothesized that some inner ear disorders may depend on a large decrease in blood pressure values associated with abnormal peripheral vasoconstriction [16]. Others reported that cochlear dysfunction could be attributed to a combination of factors including abnormal electrolytes, urea, and creatinine levels, as well as concomitant conditions such as hypertension and ototoxic medication. Antonelli et al. [10] did not find a significant correlation between BUN or serum creatinine level and hearing impairment. In experimental uremic animals, a significant decrease in Na^+^-K^+^-ATPase activity resulted in hearing impairment [17]. We tested for such correlations between hearing impairment and risk parameters with kidney dysfunction. Both serum creatinine and high blood pressure could be important independent contributing factors of disturbed cochlear function. In the presence of a low eGFR (<60 mL/min/1.73 m^2^), blood pressure treatment should be considered to preserve hearing function. Such a finding in this large-population study, performed by adjusting related important contributing factors, may be meaningful, compared with other small studies.

Ototoxic medication, including furosemide, can affect ionic gradients between the endolymph and perilymph, resulting in edema of the epithelium of the stria vascularis [18]. It is further indicated in the literature that furosemide affects cochlear function by altering the endocochlear potential [19]. In our study, we excluded 53 syndromes associated with the kidney (Alport syndrome, nephrotic syndrome, etc.) by using the questionnaire. We also excluded participants with a history of chronic renal failure. However, we could not obtain the history of use of ototoxic medication, such as NSAIDs or furosemide, as such data was not collected in this survey.

Taken together, these observations support the hypothesis of a close correlation between kidney dysfunction and cochlea impairment. The cochlea appears to be most influenced independently by CKD, which can translate into hearing impairment in adults. Although prospective studies are required to assess this association between CKD and hearing impairment, our results have considerable validity in that we confirmed this correlation in the largest population of participants to date after adjustment for other contributing factors and analyzed the study using the questionnaire and a laboratory assessment. We recommend that screening the hearing of patients with CKD to provide earlier identification of hearing impairment and earlier intervention, thereby preventing progression of hearing impairment and providing appropriate treatment strategies.

## Conclusion

The odds of hearing impairment were greater with lower eGFR than with normal eGFR. Our conclusion is that CKD combined with hypertension may be an important factor in disturbing hearing thresholds. Further studies may well reveal the common pathophysiology between kidney dysfunction and cochlear impairment.
